# Lipid metabolism of sea urchin *Paracentrotus lividus* in two contrasting natural habitats

**DOI:** 10.1038/s41598-021-93669-9

**Published:** 2021-07-08

**Authors:** Roberto Anedda, Silvia Siliani, Riccardo Melis, Barbara Loi, Maura Baroli

**Affiliations:** 1 Porto Conte Ricerche S.r.l., S.P. 55 Porto Conte, Capo Caccia, Km 8.400, Loc. Tramariglio, Alghero, SS Italy; 2 IMC-International Marine Centre, Loc. Sa Mardini, 09170 Torregrande, OR Italy

**Keywords:** Biochemistry, Chemical biology, Ecology, Chemistry

## Abstract

Sea urchins *Paracentrotus lividus* were harvested monthly from April 2015 to March 2016 from two sites in Sardinia (Italy). The two sites, a *Posidonia oceanica* meadow and a rocky bottom habitat, were naturally characterized by different food sources and availability, being mainly populated by the sea grass *Posidonia oceanica* and the brown algae *Halopteris scoparia*, respectively. Total lipids showed a minimum during winter in mature gonads, and a maximum in the summer (recovery stage). Fatty acid (FA) profiles of gut contents and gonads differed from those of the most available food sources. Levels of C18:3 (n-3) (ALA) discriminated samples from the two sites. Despite the very low amounts of C20:5 (n-3) (EPA) and C20:4 (n-6) (ARA) in *P. oceanica,* the main FA in gonads and gut contents were EPA and ARA in both sites. Increase in green algae intake prior to gametogenesis, especially *C. cylindracea*, likely affected EPA and ARA levels in gonads. The results show that *P. lividus* is able to concentrate lipids in gut contents and also to selectively store EPA, ARA and their precursors ALA and 18:2 (n-6) (LA). Moreover, bioconversion of ALA to EPA and of LA to ARA in *P. lividus* is suggested.

## Introduction

Sea urchin *Paracentrotus lividus* is naturally widespread along the European coast, both in the Mediterranean Sea and in the Atlantic Ocean^1^. A worldwide increase in market demand for sea urchin roe during the last decades of the twentieth century caused an overexploitation of this species and other edible sea urchin species. Moreover, *P. lividus* in the Mediterranean region is endangered by the "tropicalization" process^2^ related to fast warming (from 0.24 °C decade^−1^ west of the Strait of Gibraltar to 0.51 °C decade^−1^ over the Black Sea)^3^. Consequently, nowadays a growing attention to sustainable fishing is being paid, while at the same time increasing efforts are directed to the improvement of sea urchin breeding strategy. To this end, stock enhancement programs are promoted while meeting market demand^4^.\

In order to improve echinoculture practices and support sustainable harvesting programs, a solid knowledge of the metabolic processes affecting sea urchin growth and reproduction is considered an asset^5–8^. Considerable efforts have been spent over the years in this direction; some studies on echinoderms, also specifically directed to *P. lividus*, have been undertaken to deepen the understanding of sea urchin metabolism^9,10^, in particular concerning their reproductive cycle and factors affecting it^8,11–13^. Given the high commercial value of sea urchin roe, a number of studies focused on the identification of the most important factors affecting the chemical composition of gonads^14–22^. Our previous investigations on the lipid composition of sea urchin gonads demonstrated that both physiological and seasonal factors considerably affect lipid absorption and storage mechanisms. Moreover, they highlighted the marked influence of some environmental parameters on fatty acid profiles^21,22^. However, the complex interrelationship between environmental or seasonal factors, food availability, diet composition, feeding habits and molecular composition of *P. lividus* gonads has not been fully understood yet, especially in natural habitats. Previous investigations have been carried out in order to understand the complex role of different habitats on population structure and the biological conditions of sea urchins *P. lividus*^23^ but their effects at molecular level on lipid profiles are less known.

*Paracentrotus lividus* is an herbivorous echinoid which feeds preferably on live macrophytes or macroalgae: its food preferences have been already characterized^1,24–26^. The main purpose of the present paper is to describe *P. lividus* nutritional metabolism, with a specific focus on the effect of two marine habitats mainly populated by a sea grass (*Posidonia oceanica*) and a macroalga (*Halopteris scoparia*) constituting sea urchin diet in the selected natural environments^1,25^. Moreover, we discuss the impact of dietary fatty acids on the gonadal fatty acid profiles over a complete seasonal cycle.

## Results

In the present work, both total lipid content and fatty acid profiles of the most abundant sea grass and macroalgae, gonads and gut contents of sea urchin *P. lividus* were analyzed as a function of time over one year. In particular, the aforementioned analyses were performed on samples monthly collected from two habitats, namely the rocky bottom site, mainly populated by the brown macroalgae *Halopteris scoparia*, and a *Posidonia oceanica* meadow.

The *P. oceanica* meadow was approximately 400 m far away from the nearest rocky substrate. The presence of sand and dead matte between the two sites, which are devoid of food and easily expose echinoids to predators, suggests that sea urchins collected in one site were not affected by dietary sources from the other site.

### Natural diets

*Halopteris scoparia* visibly covered the largest part of the rocky bottom. Dietary sources other than *H. scoparia* are reported in Table S1 (“Supplementary Material”). Other minor species populating the rocky bottom such as *Dictyota dichotoma, Padina pavonica, Halimeda tuna*, and other encrusting corallinaceae, also possible sources of lipids in sea urchins diet, have quite low lipid content (2–7% D.W.).

In the *Posidonia oceanica* meadow site, many taxa were identified, including red, brown and green algae (especially *Caulerpa cylindracea*) and also small animals. *P. oceanica* constituted more than 46% of the gut content on average (with a peak at 63%), being by far the most represented species in the gut of *P lividus* in that site, followed by (not coralline) turf (annual mean 21.0 ± 9.5%) and other species (annual mean < 10%). All other species always constituted less than 10% of the gut content, except turf (not coralline turf), which constituted about 21%, on average. Green algae increased in gut content in the period preceding gametes maturation (October-December). Animal taxa represented, on average, ~ 2% of the gut content in *P. oceanica* meadow (Table S2, Fig. S1, “Supplementary Material”).

### Total lipid content in gonads and gametogenic stages

**Figure 1:**
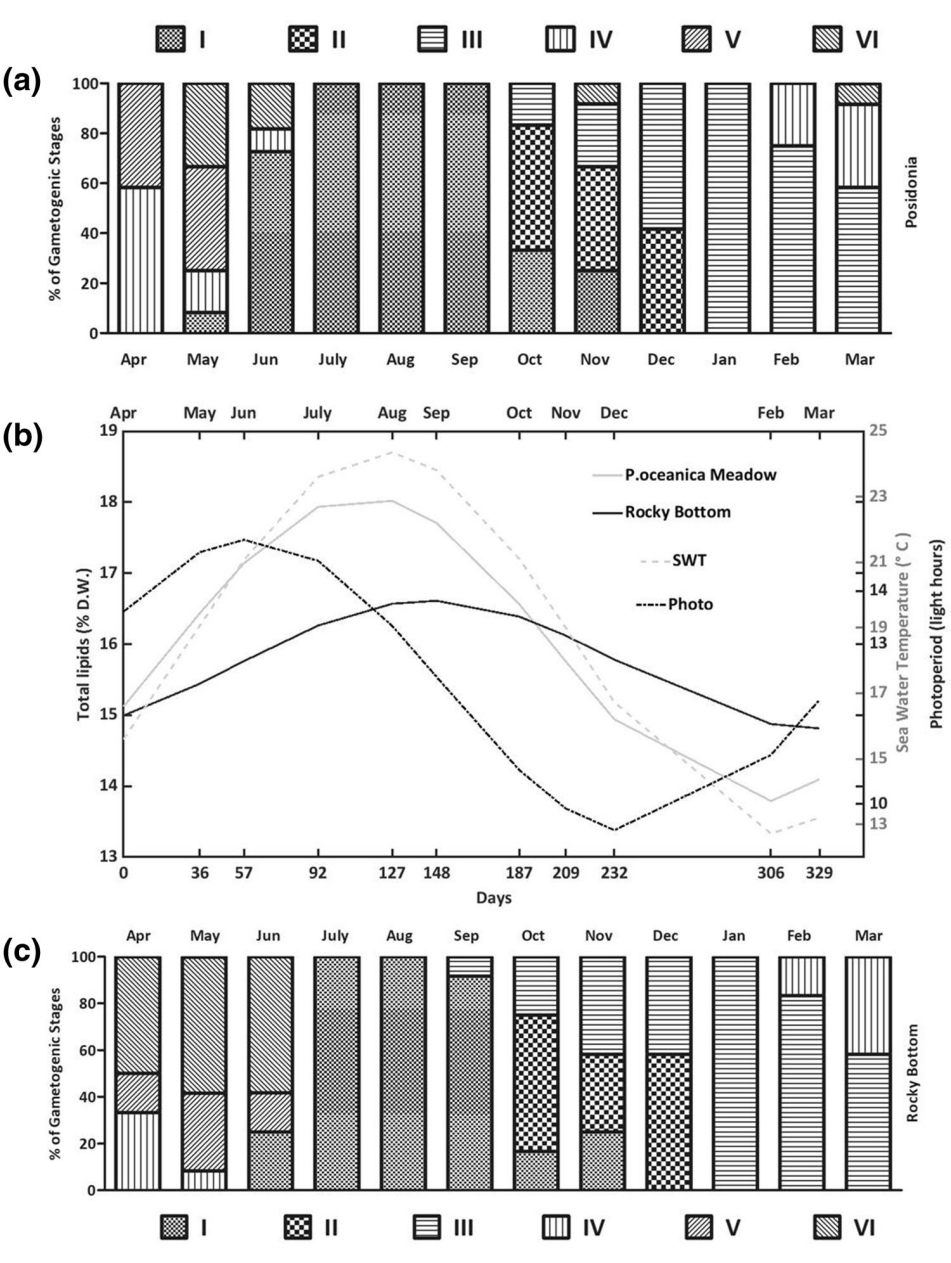
Seasonal variation of total lipids in gonads of *P. lividus* from *P. oceanica* meadow and rocky bottom habitats, compared with fitted photoperiod and seawater temperature. (**a**) gametogenic stages detected in the *P. oceanica* meadow. Fitting in (**b**) was performed according to Siliani et al.^22^ (**c**) gametogenic stages detected in the rocky bottom habitat.

Figure 1 describes the observed changes in total lipid content of gonads of *P. lividus* harvested in the two habitats (i.e. the rocky bottom site and *P. oceanica* meadow site), together with seasonal fluctuations of temperature, light hours and gametogenic stages detected.

The seasonal variation of gonad total lipids can be described by the mathematical model previously proposed by Siliani et al*.*^22^ (Table S3, “Supplementary Material”). Briefly, changes in the lipid content of gonads followed a change in photoperiod in both sites, while they appeared to be less clearly correlated to changes in temperature, in agreement with previous observations^22^. Moreover, lipid content changed to a larger extent (i.e. reached a higher maximum and a lower minimum) in specimens from the *P. oceanica* meadow than from the rocky bottom site. A maximum in total lipids was found, in both sites, between August and September, when a prevalence of gametogenic stage I (recovery) was observed. Minimum lipid contents were detected in the period February–March–April, corresponding to the presence of a large amount of mature gametes (stages III, IV and V, according to the nomenclature proposed by Byrne^27^).

**Figure 2:**
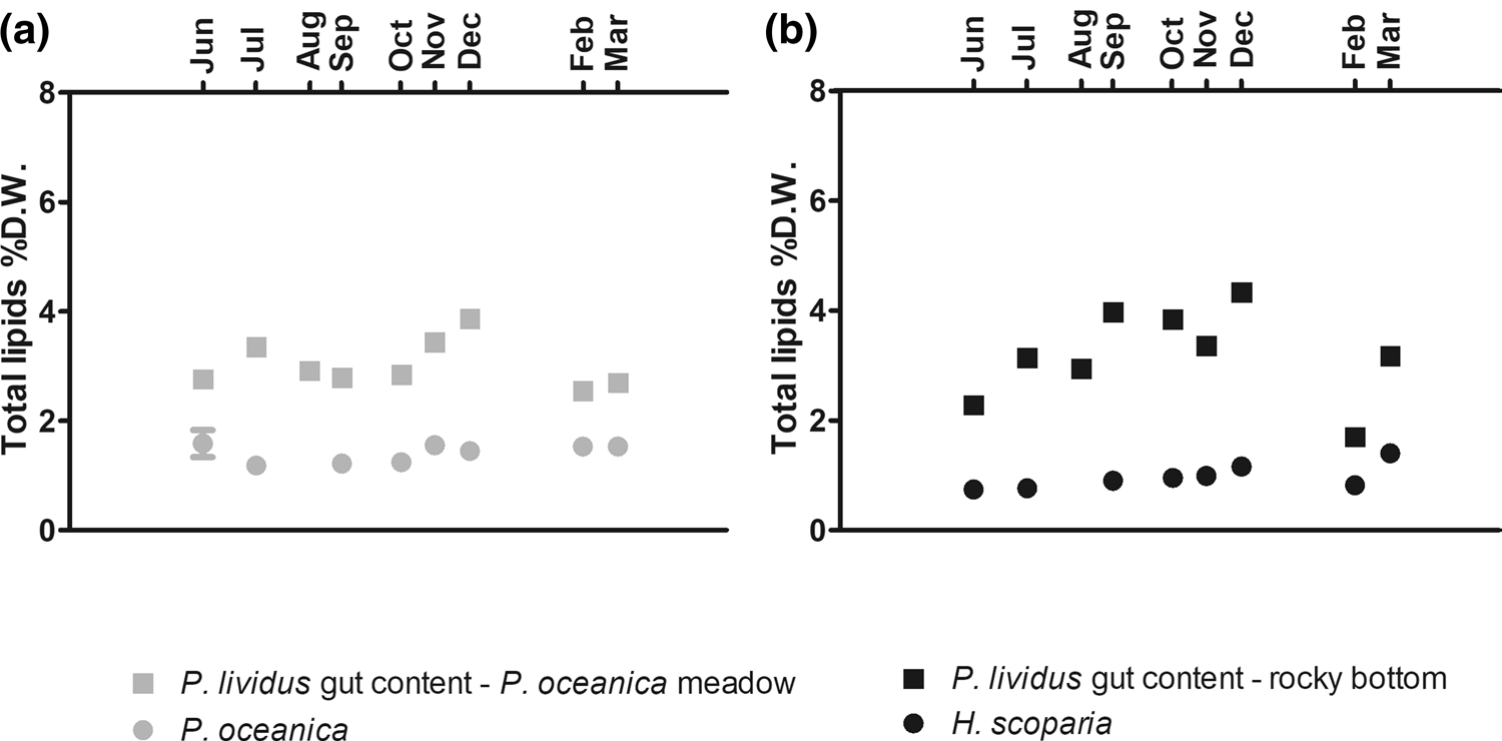
Seasonal variation of total lipids content in *P. oceanica* and *H. scoparia* from *P. oceanica* meadow (**a**) and rocky bottom (**b**), respectively. Each point corresponds to the analysis of a pooled sample, as detailed in “Materials and methods” section.

Figure 2 shows that total lipid content of *P. oceanica* and *H. scoparia* was very low, about 1–2% D.W. and that seasonal fluctuations were almost negligible in the sea grass and macroalgae populating the two sites. It is also clear that total lipids in the gut showed higher values than the sea grass and macroalgae analyzed. Regardless the habitat, lipid level in the gut content was, on average, approximately four times lower than in gonads (Fig. 1, Fig. 2).

### Comparison of fatty acid profiles: mean annual values

**Figure 3:**
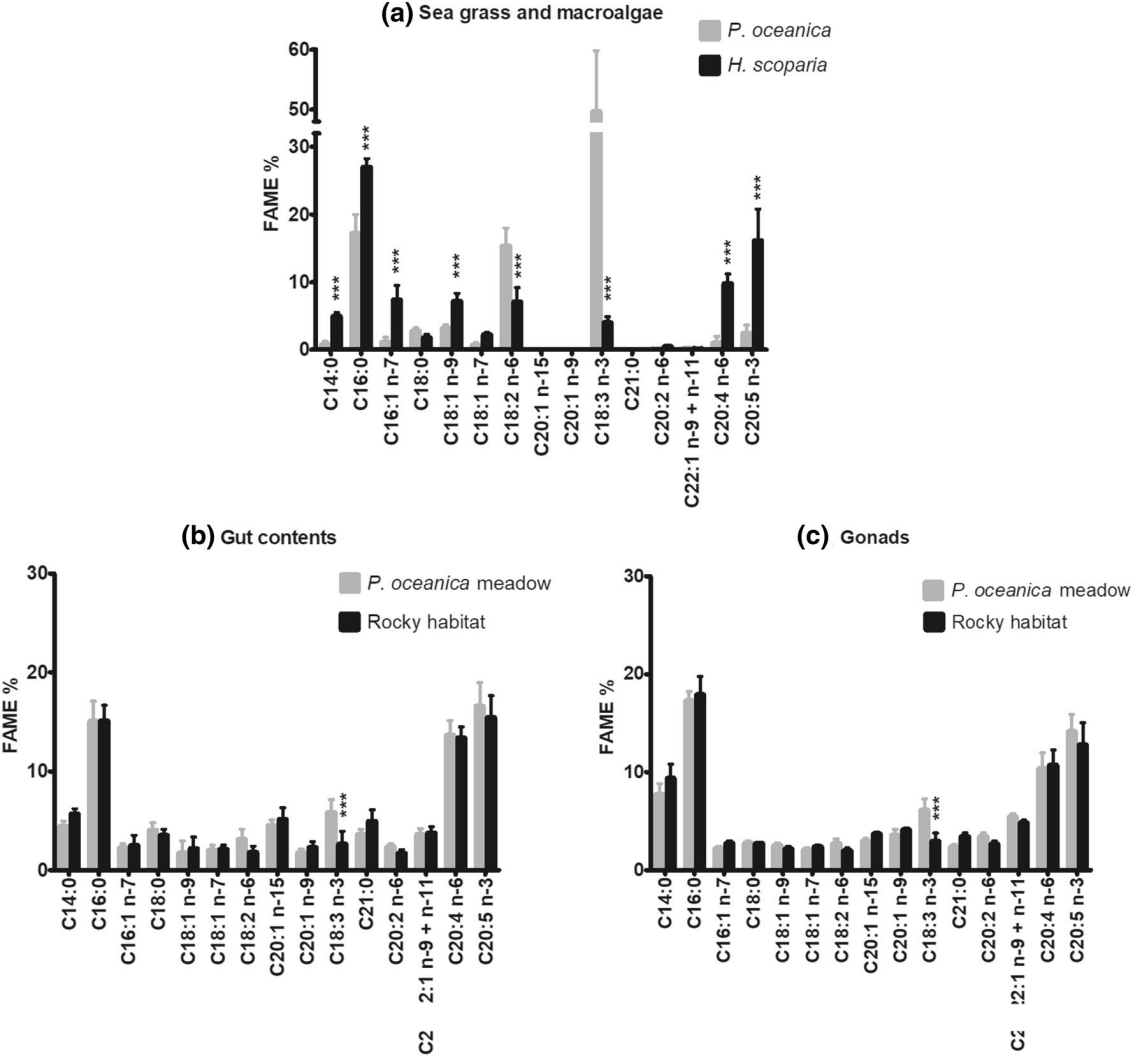
Fatty acid profiles (annual mean values, % with SD) of *P. oceanica* and *H. scoparia* (**a**), *P. lividus* gut contents (**b**) and gonads (**c**) from *P. oceanica* meadow and rocky bottom. Associated asterisks *, **, *** indicate significant differences between the two areas for each fatty acid, with P < 0.05, P < 0.01 and P < 0.001, respectively.

The mean annual values of the most representative fatty acids of *P. oceanica* and *H. scoparia*, gut contents and gonads of *P. lividus* are shown in Fig. 3.

#### Fatty acid profiles of *P. oceanica* and *H. scoparia*

Fatty acid composition of the main sea grass and macroalgae which populated the two sites appeared markedly different from each other (Table 1), with highly significant (P < 0.001) differences of several fatty acids (Fig. 3). PUFA was the most represented fatty acid category in both dietary substrates, followed by SFA and MUFA. This was observed all year round (Fig. S3). As a matter of fact, the main differences between fatty acid profiles from *P. oceanica* and *H. scoparia* generally concerned the PUFA class. The main PUFA detected were of the C18 and C20 groups in both species. In particular, *H. scoparia* showed higher C 20:5 (n-3) and C 20:4 (n-6) content, whereas *P. oceanica* was characterized by much higher levels of C 18:3 (n-3) and higher content of C 18:2 (n-6) (Fig. 3). The most significant difference between the lipid profiles of *P. oceanica* and *H. scoparia* involved C 18:3 (n-3); this FA was dominant in *P. oceanica*, with a mean value of 49.65% ± 10.16, whereas it reached a mean value of 4.03% ± 0.87 in *H. scoparia* (Fig. 3 and Table 1). C 16:0 was the main SFA, while C 16:1 (n-7) and C 18:1 (n-9) mainly represented the MUFA category in both *P. oceanica* and *H. scoparia*.

**Table 1:** Fatty acid profiles (annual mean values, % with SD) of *P. oceanica* and *H. scoparia*.

FAME	*P. oceanica*	*H. scoparia*
C14:0	0.77 (0.46)	4.94 (0.58)
C16:0	17.20 (2.80)	27.01 (1.23)
C16:1 n-7	1.12 (0.71)	7.35 (2.20)
C18:0	2.73 (0.49)	1.80 (0.47)
C18:1 n-9	3.16 (0.53)	7.16 (1.15)
C18:1 n-7	0.70 (0.34)	2.23 (0.30)
C18:2 n-6	15.31 (2.67)	7.06 (2.12)
C18:3 n-3	49.65 (10.16)	4.03 (0.87)
C20:2 n-6	0.14 (0.09)	0.50 (0.12)
C22:1 n-9 + n-11	0.17 (0.03)	0.10 (0.04)
C20:4 n-6	1.05 (0.94)	9.77 (1.43)
C20:5 n-3	2.44 (1.23)	16.14 (4.65)

#### Fatty acid profiles of gonads and gut contents

Although *P. oceanica* and *H. scoparia*, the most largely available dietary sources in the two habitats, significantly differed for several fatty acids, as summarized in Fig. 3, the most impacting on sea urchin composition was C 18:3 (n-3). A highly significant difference (P < 0.001) between the two habitats was ascribed to 18:3 (n-3) in both gut contents and gonads. Multivariate statistical analyses of the experimental data confirmed the same conclusion (Table S4, “Supplementary Material”). As expected, 18:3 (n-3) was higher in sea urchins from *P. oceanica* meadow, thus reflecting the difference between the fatty acid profiles of the main dietary substrates. Differences in other diet-related fatty acids, such as C 18:2 (n-6), C 14:0 and C 16:0, were not statistically significant (P > 0.05) in sea urchin gut and gonads.

Similarly, it clearly appears from Fig. 3 that the significant differences in C 20:5 (n-3) and C 20:4 (n-6) found in the selected dietary sources were not reflected in gut contents and gonads of *P. lividus*. Interestingly, despite *H. scoparia* contained, on average, more C 20:5 (n-3), this FA was found in slightly higher proportion in both gut and gonads of specimens collected from *P. oceanica* meadow than in samples collected from the rocky bottom habitat. On one hand, these observations may suggest that other dietary sources of fatty acids might be meaningful; on the other hand, they highlight the need to further investigate the metabolic mechanisms implied in fat storage and utilization in *P. lividus*, since C 20:5 (n-3) and C 20:4 (n-6) may be likely produced by biosynthetic processes from other essential fatty acids*.*

### Seasonal variation of fatty acid profiles

*Paracentrotus lividus* gonads were characterized by a high proportion of PUFA, followed by SFA and MUFA (Fig. S3) year round.

Gut contents showed quite similar fatty acid composition in both sampling sites, but did not reflect the lipid composition of *P. oceanica* and *H. scoparia*.

Increase of PUFA in gut content corresponded to a decrease in SFA and anticipated similar variations of the same fatty acid categories in gonads. While a seasonal effect was evident for PUFA and SFA in both gonads and gut contents, MUFA were almost stable throughout the year. Especially in the rocky bottom habitat, gonadal PUFA exhibited a considerable decrease throughout spring and summer, going from 42.29% ± 0.08 in April to 34.39% ± 0.66 in August. In all cases, PUFA decrease appeared to be well balanced by a corresponding increase in SFA. The observed changes in lipid categories were likely driven by specific fatty acids, as explained in the following.

### Seasonal variation of C 18:3 (n-3) and C 18:2 (n-6)

**Figure 4:**
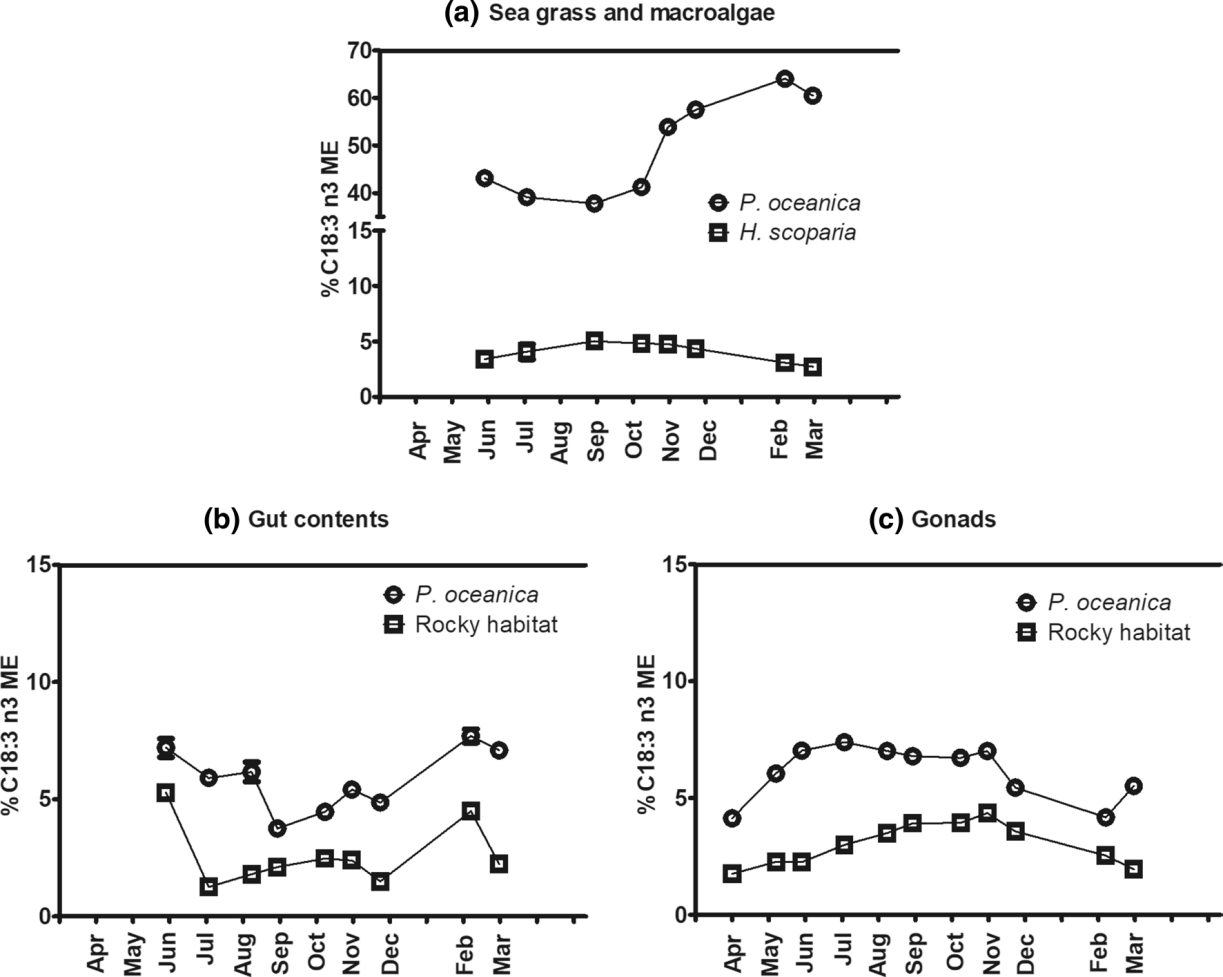
Seasonal variation of C 18:3 (n-3) in *P. oceanica* and *H. scoparia* (**a**), gut contents (**b**) and gonads (**c**) of *P. lividus* collected from *P. oceanica* meadow and rocky bottom. Each point corresponds to the analysis of a pooled sample, as detailed in “Materials and methods” section.

A detailed description of the seasonal fluctuations of C 18:3 (n-3) levels in the analyzed sea grass and macroalgae, gut contents and gonads of *P. lividus* is reported in Fig. 4.

C 18:3 (n-3) was always higher in the samples from *P. oceanica* meadow. In rocky bottom, it increased from April to November in gonads, reaching a maximum of ~ 4%, whereas in the *P. oceanica* meadow this percentage increased from April to July, then reached a plateau of about 7% and a decrease started from November, when sea urchins metabolism was mainly influenced by production of gametes and gonads reached premature/mature stages (Fig. 1)^22^.

**Figure 5:**
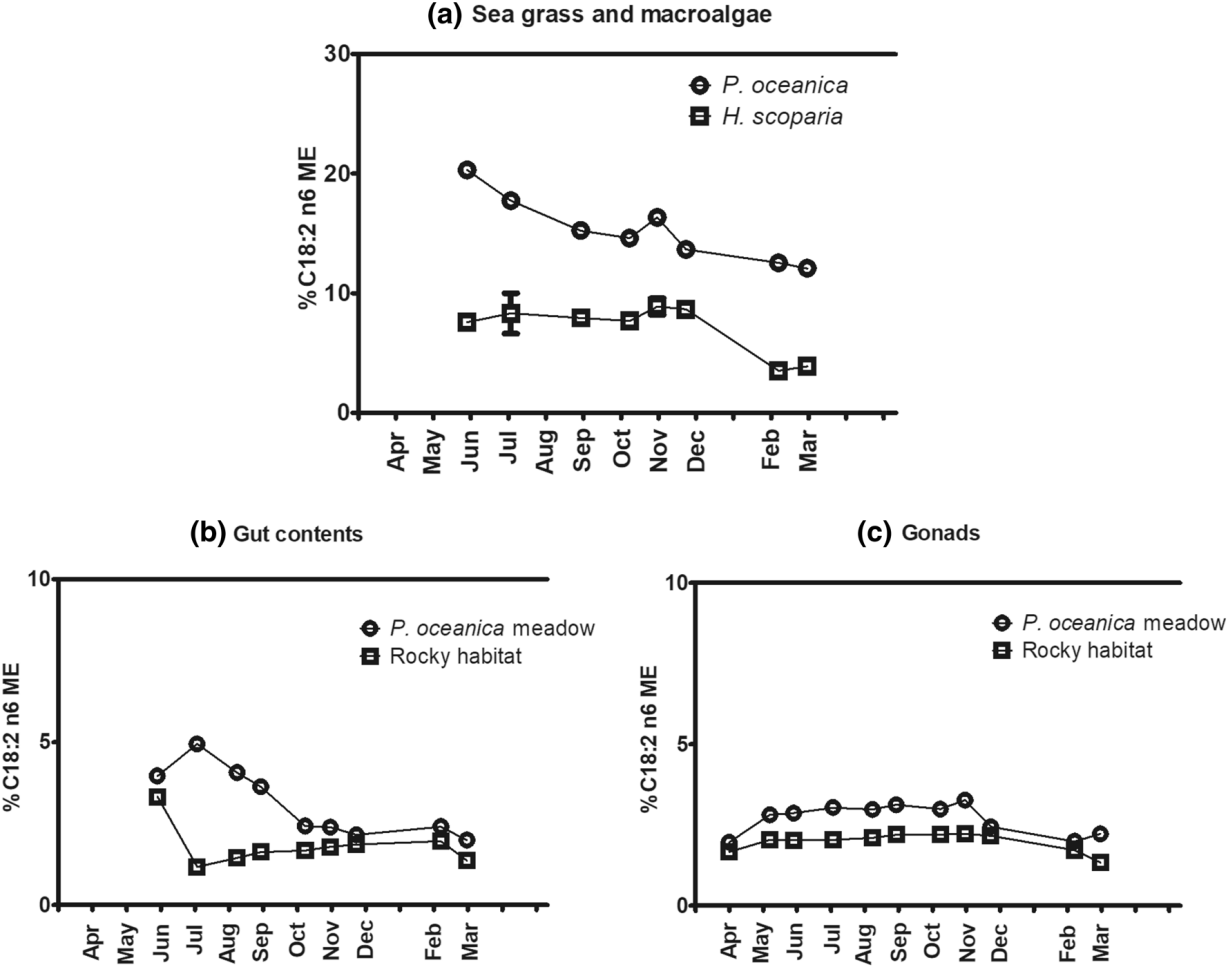
Seasonal effect on C 18:2 (n-6) contents in sea grass and macroalgae (**a**), gut contents (**b**) and gonads (**c**) of *P. lividus* from the two sites (*P. oceanica* meadow and rocky bottom). Each point corresponds to the analysis of a pooled sample, as detailed in “Materials and methods” section.

Similarly, C 18:2 (n-6) always showed higher abundance in specimens from the *P. oceanica* meadow than from the rocky site (Fig. 5).

### Effect of season on C 20:5 (n-3) (EPA) and C 20:4 (n-6) (ARA)

**Figure 6:**
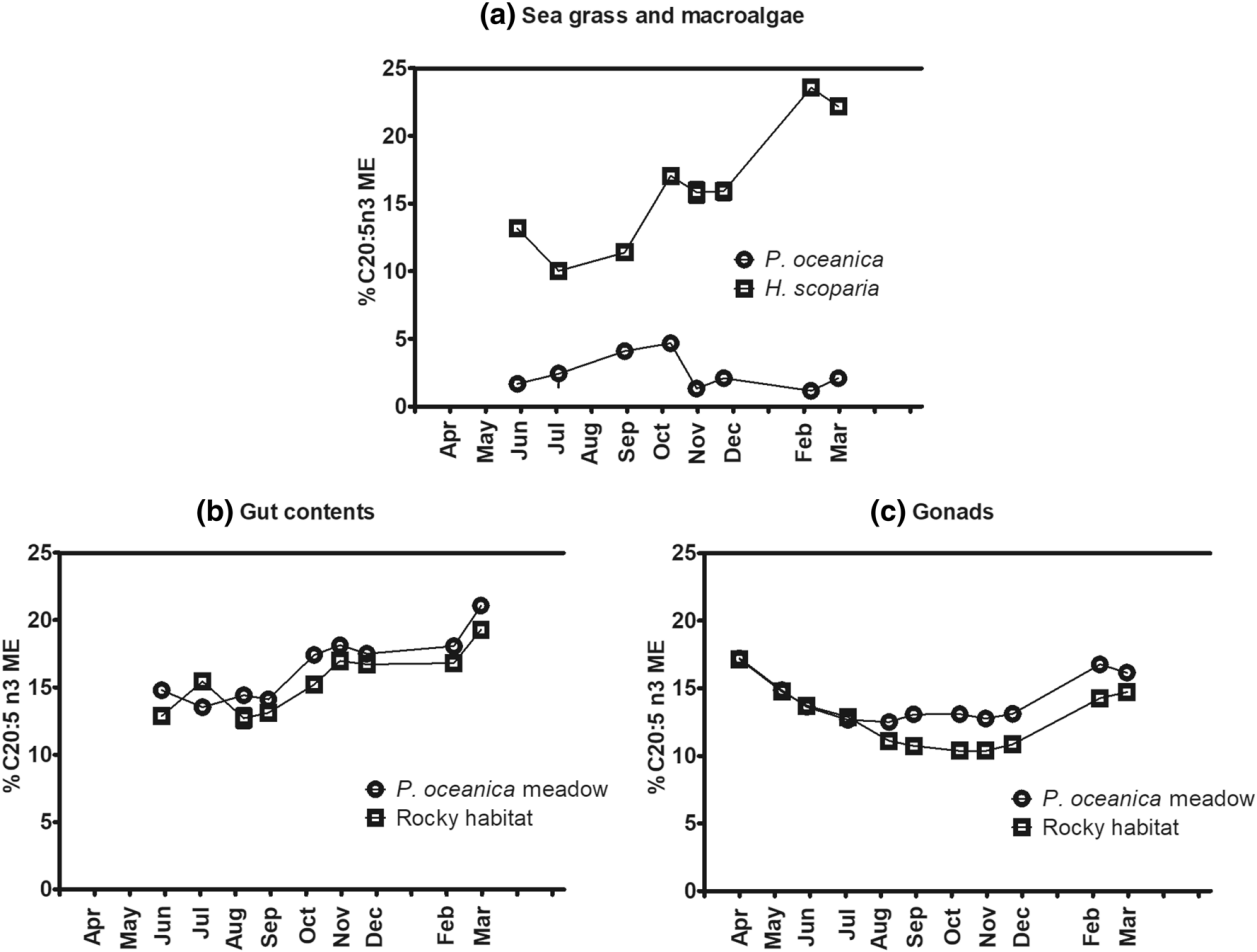
Seasonal variation of C 20:5 (n-3) in the most representative dietary sources (**a**), gut contents (**b**) and gonads (**c**) of *P. lividus* from *P. oceanica* meadow and rocky bottom. Each point corresponds to the analysis of a pooled sample, as detailed in “Materials and methods” section.

The main PUFA in the lipid fraction of gonads and gut contents, regardless the growing habitat (i.e. the diet), were C 20:5 (n-3) and C 20:4 (n-6) (Fig. 3), in agreement with previous reports^14,15,19–22^. C 20:5 (n-3) and C 20:4 (n-6), together with C 16:0, were the most abundant in both gut and gonads. Interestingly, although C 20:5 (n-3) contents in the sea grass and macroalgae analyzed were always very different from each other (Fig. 6), levels of this FA in gut contents and gonads followed analogous trends in the two habitats.

**Figure 7:**
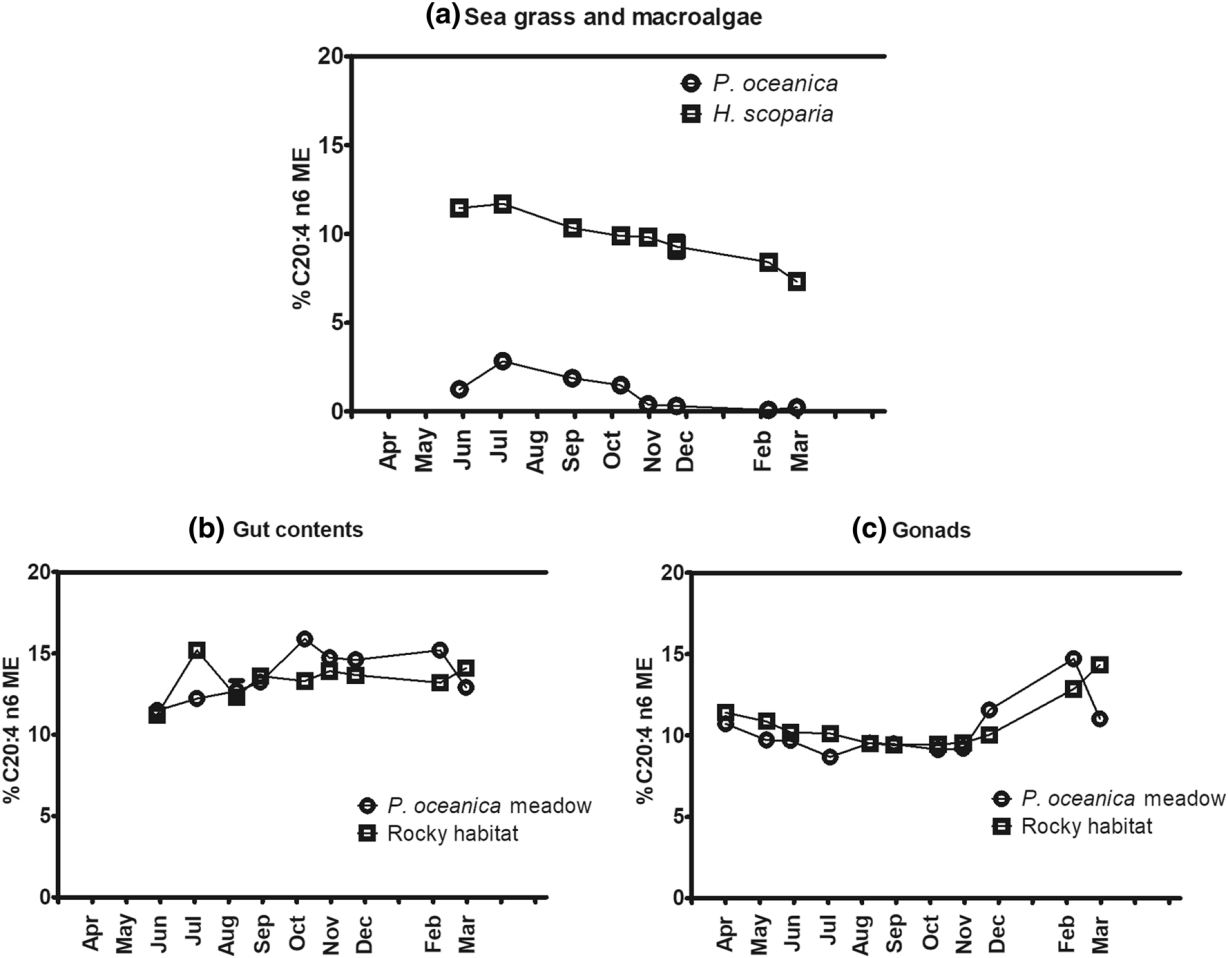
Seasonal variation of C 20:4 (n-6) in the sea grass and macroalgae analyzed (**a**), gut contents (**b**) and gonads (**c**) of *P. lividus* from the two sites analyzed (*P. oceanica* meadow and rocky bottom). Each point corresponds to the analysis of a pooled sample, as detailed in “Materials and methods” section.

A similar behaviour was observed for C 20:4 (n-6) (ARA) (Fig. 7). C 20:4 (n-6) levels in gonads showed similar trends in both habitats, but important differences were especially observed from December to March. Despite in the period from December to February C 20:4 (n-6) was almost absent in *P. oceanica* (0.09% ± 0.01–0.31% ± 0.01), gonads in the *P. oceanica* meadow contained more of this FA than in the rocky habitat.

C 20:5 (n-3) and C 20:4 (n-6) were the most concentrated FA of *H. scoparia* after C 16:0, representing 16.14% ± 4.65 and 9.77% ± 1.43, respectively, on an annual mean basis. In contrast, *P. oceanica* was characterized by much lower contents of C20:5 (n-3) and C 20:4 (n-6) (2.44% ± 1.23 and 1.05% ± 0.94, respectively, annual mean values) (Table 1 and Fig. 3).

The stomach content of the urchins harvested from the *P. oceanica* meadow contained green algae, especially *C. cylindracea*, all over the year, particularly in the fall/winter season (October-December) (Table S2, Fig. S1). During fall (October-December), C 20:5 (n-3) and C 20:4 (n-6) content in gonads were the lowest. In January, green algae in *P. lividus* gut was reduced to 6.5%, which further reduced to 4.5% in February, when C 20:5 (n-3) content in gonads was found to increase considerably. However, C 20:5 (n-3) and to a less extent C 20:4 (n-6), increased in gut contents from October, likely reflecting the increased dietary intake of green algae. Brown algae in the *P. oceanica* meadow site, which included *H. scoparia*, were scarcely present in sea urchin diet from July on (~ 5% or less).

## Discussion

Results of our research showed that lipid accumulation in sea urchin gonads follows a periodic fluctuation, in agreement with previous observations^22^. The analysis of Fig. 1 suggests the key role of photoperiod in triggering and then modulating fat utilization and storage mechanisms in *P. lividus* gonads, while the effect of temperature in gametogenesis and spawning in echinoderms still remains uncertain^5,22,28^. In fact, a change in photoperiod anticipated the corresponding change in gonad total lipids content in both habitats, while the role of temperature was not very clear, since lipid changes seemed not to be associated with changes in temperature. Most likely, the combined effect of both parameters regulates reproductive cycle of sea urchins. Similar periodical trends in total lipid content were also observed in recent studies on *P. lividus* gonads^19,20^ collected in different geographical areas. For example, Rocha et al.^20^ reported that gonadal lipid content are likely influenced by the environmental conditions characterizing the harvest site in the Praia Norte (Portugal). In this work and in the abovementioned studies, total lipid content in gonads changed as a function of gametogenic cycle, i.e. increased until the recovery/growing stage (I–II) and then progressively decreased until the premature/mature stage (III–IV)^27^. In another detailed characterization of *Arbacia dufresnii*, Dìaz de Vivar et al.^29^ observed a marked dependence of the total lipid content with gonad maturation, with a significant decrease in lipid content in spawned compared to intact gonads, especially in female sea urchins.

Assuming *P. oceanica* and *H. scoparia* were the main dietary sources of lipids in our study, gonad lipid content was relatively independent from dietary lipid intake, in agreement with data from other authors^19,20,22^. Indeed, total lipids in *P. oceanica* and *H. scoparia* were very low (approximately 1% D.W.) and seasonal variations of lipid levels in these main dietary substrates were definitely negligible. These results are further supported by the literature^30,31^. For a better understanding of the comparison between different scientific reports^19,20,22^, it should be recalled here that the displacement of periodical gametogenic cycles is strongly influenced by several environmental factors and is, therefore, dependent on the growing habitat^16,32^.

As far as the commercial value of sea urchin gonads is considered, several reports^20,33^ suggest that the best harvesting period is when gonads are in the growing stage, when nutrient contents (i.e. proteins, lipids and carbohydrates) are at their highest levels, and when sensorial characteristics are optimal. In fact, gonad maturation decreases the overall quality of roe, and make them more bitter and less pleasant^33,34^. However, it is striking that very often the official regulation on the harvest of *P. lividus* in Sardinia allowed collection of sea urchins in the period from November to April, when products are nutrient-poor and in the late stages of gametogenesis (i.e. pre-mature, mature and spawning stages)^35^.

Our observations suggest that total lipids from dietary sources concentrate in the gut. The amount of lipids in these latter samples is actually always much higher than in the sea grass and macroalgae analyzed. The concentration of lipids in the gut has been already observed in other echinoderms as well^36,37^. These evidences suggest that digestion phenomena occurring in the gut may include the concentration of nutrients. Moreover, our data show that lipid fatty acid composition in gut is considerably consistent, regardless dietary lipid. While further studies are needed, most recent findings strongly suggest that gut flora have a role in assisting digestion and absorption of nutrients in sea urchins^38^. De novo synthesis of fatty acids by microbiotes, an interesting hypothesis that would especially concern the modulation of short chain fatty acids levels, should be further and specifically investigated. Based on most recent findings, a possible role of bacteria in nutrient production and processing has been postulated^39^. However, it should be also reckoned that other lipids may come from other dietary sources beyond the main sea grass and macroalgae (“Supplementary Material”). This latter hypothesis, however, would not explain the substantial increase observed in gut lipids, since other possible sources do not have very high lipid contents and were taken in small percentage. For example, it was previously observed in adult *Strongylocentrotus intermedius* that algal pellets exceeded 80–90% (wet weight) of gut contents, complemented by detritus, small animals (e.g. small crustaceans and mollusks) and non-foods (e.g. sand, shell fragments)^40^. Moreover, in *P. lividus* sampled from natural conditions in Corsica (France), 95% of the total gut content was represented by plant material^41^. Similarly, animal taxa in our study represented a very low percentage of the gut content, and species populating the rocky bottom, other than *H. scoparia*, have low lipid content and likely had little relevance on sea urchins diet. Also Murillo-Navarro and Jimenez-Guirado^25^, in a yearlong investigation, found that *H. scoparia* was the most abundant brown alga in gut contents of *P. lividus*.

Brown algae and leaves of *P. oceanica* are in fact generally considered among the primary components of adult *P. lividus* diets^1,24,25^. It has been also observed by other authors that sea urchins consume all parts of *P. oceanica* and preferentially green leaves colonised by epiphytes^1,26,42–44^. Epiphytes were not removed from our samples before analysis.

The role of gut and stomach as nutrient storage organs is generally acknowledged^41,45^. This is demonstrated by the almost double lipid contents found in gut than in food sources in the present investigation and by other studies^36,37^. As a later digestion step, lipids are selectively stored in gonads, where almost three or even four times the lipids contents found in the gut were detected. This supports the hypothesis of lipid relocation from gut to gonads, thus confirming the role of gonads as an important storage tissue for *P. lividus*, as was previously established by other authors^22,46^ and correspondingly a role in lipid metabolism can be ascribed to the digestive tract. It also further proves that the amount of fat daily introduced with diet has only a limited influence on the seasonal evolution of total lipids in gonads. Of course, nutrients and especially lipids stored in gonads serve during gametogenesis, as an energy source for developing embryos and are mobilized during pre-feeding development of larvae^5^. In echinoderms, indeed, nutrients provided in the eggs are needed by developing embryos and larvae.

In two recent investigations on *P. lividus* collected along the Atlantic coast of Portugal, Rocha et al.^19,20^ evidenced slightly different seasonal trends. They observed both a maximum lipid content and an increase in PUFA content in gonads during the fall season. In contrast, we observed a peak in total lipids during summer, and an increase in PUFA during winter. Likely, the different climatic and environmental conditions of the Atlantic coast with respect to the Mediterranean basin (especially seawater temperatures) induce different gametogenesis cycles^16^, which in turn modulate the lipid balance in gonads. Gametogenic stages are in fact differently distributed along the year in ours and the cited works by Rocha et al.^19,20^. In general, lipid content in gonads seem to increase during the recovery (stage I) and growing (stage II) gametogenic stages^27^, when gonads are packed with nutritive phagocytes and only few germ cells are present.

Other studies suggested that specific fatty acids found in the gonads of sea urchins may be synthesized by other tissues such as the intestine and then mobilized to the gonads^47^.

Regardless the different food availability in the two analyzed sites, our results show a remarkable robustness of the fatty acids profile of gut contents. This is particularly interesting since they show a regulation of physiologically essential C 20:5 n-3 and C 20:4 n-6 at gut level, which seem to quite finely level out according to season, regardless the dietary contents of these fatty acids.

The increase in gonad PUFA observed in both habitats during winter did not seem to correlate with substantial changes in the main taxa isolated in the gut content of the sea urchin sampled in the *P. oceanica* meadow, nor to relevant changes in the specimens populating the rocky bottom habitat (“Supplementary Material”). This is consistent with our previous studies^21,22^, which linked the phenomenon to both the cold acclimatization effect and gametogenesis. Raise in PUFA in lower temperatures allows maintaining cell membrane fluidity and, consequently, supports its functionality.

The questions arise whether the lipid species contained in the food sources can be directly and selectively absorbed by sea urchin gonads and how much food habits affect gonads composition. In order to answer these questions, discussion should be directed to each relevant fatty acid.

The fatty acids of glycerolipids of higher-plants chloroplasts are highly unsaturated, and the most represented fatty acid is C 18:3 (n-3)^48^. Instead, brown algae, such as Phaeophyceae, contain a large amount of C 20:4 (n-6) and C 20:5 (n-3)^49^. During our studies, the most significant difference between the fatty acid profiles of *P. oceanica* and *H. scoparia* was related to C 18:3 (n-3). According to our data, *P. oceanica* contained, on average, more than ten times the amount of this FA in *H. scoparia*.

The fatty acid profile of *P. oceanica* described in the present study is in agreement with previous reports^50,51^ and confirms that lipids of *P. oceanica* are mainly represented by the C 18:3 (n-3), C 18:2 (n-6), and C 16:0^51^. On the contrary, the fatty acid composition of *H. scoparia* seems to be quite variable considering previously published reports, although literature generally agrees on the most abundant fatty acids (i.e. C 16:0, C 18:2 n-6, C 20:5 n-3 and C 20:4 n-6)^31,52^.

Both in rocky bottom and in *P. oceanica* meadows, gonadal C 18:3 (n-3) decreased when sea urchins metabolism is mainly influenced by production of gametes (from November), i.e. when gonads reached premature/mature stages, as previously observed^15,22^. Our data showed a decrease of C 18:3 (n-3) in gut roughly corresponding to an increase of the same FA in gonads (Fig. 4), suggesting that dietary C 18:3 (n-3) was not selectively and directly retained in gonads from the diet, but likely took active part to metabolic processes of bioconversion or is catabolized during β-oxidation of lipids.

Also C 18:2 (n-6) showed a similar behaviour in our study and in other previous investigations^15,20^.

Remarkably, C 20:5 (n-3) and C 20:4 (n-6) were the most abundant LC-PUFA in both gut and gonads, in contrast with the composition of the main dietary sources of lipids in the two habitats. In fact, while high percentages of these fatty acids were found in the brown algae *H. scoparia*, they were present only in very low percentages in the *P. oceanica* samples. In sea urchins, the fatty acid profile of diet is often scarcely reflected in gut contents and gonads^53^. From July to March we detected higher percentages of C 20:5 (n-3) in gonad samples collected from *P. oceanica* meadow than in the corresponding samples from rocky bottom. Moreover, our data clearly show that the C 20:5 (n-3) contained in either gonads and gut does not reflect seasonal variations of this FA in the main sea grass and macroalgae populating the two sites. This result supports earlier observations^5,21^.

Beyond *P. oceanica*, green algae, especially *C. cylindracea*, represented additional dietary sources of C 20:5 (n-3) in the *P. oceanica* meadow. *P. lividus* usually feeds on brown algae and only less frequently on green algae^1,15^. In fact, green algae represented less than 5% of the gut content in *P. oceanica* meadow all year long but from October to December, when they increased from 10 to 25%. In this period, C 20:4 (n-6) and C 20:5 (n-3) in gonads reached their lowest values, but the C 20:5 (n-3) content in gut noticeably increased. After January, when sea urchin reduced feeding in green algae and again less than 5% of green algae was found in the gut content, C 20:5 (n-3) and C 20:4 (n-6) content in sea urchins gut started increasing. To explain this observation, we recall that it was found in *S. droebachiensis* that dietary FA were not incorporated in sea urchin tissues after short feeding experiments^54^, but longer experiments allowed to observe diet-related modifications in tissues^36^. Therefore, it is reasonable to think that nutrients are transferred from gut to gonads. Among other dietary sources of lipids, brown algae in *P. oceanica* meadow likely did not significantly contribute to increase LC PUFA in gut contents and gonads prior to gametogenesis, being brown algae intake almost always low in the present study.

The observed increase of C 20:4 (n-6) in gonads in December was less correlated to the dietary availability of this FA, but was likely associated to cold adaptation and to the growth and maturation of gametes^21^. In fact, even when the main dietary source of lipids, *P. oceanica*, was almost completely devoid of this FA, the percentage of C 20:4 (n-6) in gonads was 10–15% and not significant increase of this FA was observed in gut contents from October to December.

As for most aquatic consumers, C 20:5 (n-3) and C 20:4 (n-6) can be selectively retained in gonads from dietary sources or accumulated through the conversion of other essential 18-carbon FA.

Since we found similar amount of C 20:5 (n-3) C 20:5 (n-3) and C 20:4 (n-6) in *P. lividus* gonads and gut contents and these values were much higher than in dietary sources, retention or biosynthesis should have occurred already at intestinal level, as previously suggested for other echinoderms^36,37,47^. As previously hypothesized for *Strongylocentrotus intermedius*, likely these FA were transferred to gonads after being processed and stored in the digestive tract^47^.

Recently, Kabeya et al*.*^55^ found that *P. lividus* possesses desaturases that are able to convert C 18:3 (n-3) and C 18:2 (n-6) into C 20:5 (n-3) and C 20:4 (n-6), respectively. Han et al*.*^47^ characterized the expression of fatty acid desaturases (*SiFad1*) in different tissues of *S. intermedius* and concluded that the highest expression is in the intestine, while gonads have lower expression level. Therefore, while retention from diet and biosynthesis from C_18_ precursors of essential lipid species such as C 20:5 (n-3) and C 20:4 (n-6) might occur already in the gut^36,37,41,45^, also gonads might possess some, likely lower, biosynthetic functions. Kabeya et al*.*^55^ did not specifically quantify the expression of desaturases in different tissues of *P. lividus*, therefore further research in this sense would be beneficial.

It should be mentioned that sex-induced difference of fatty acid profiles of sea urchin gonads were not studied in the present work, but males and females specimens were pooled together. Some previous reports have evidenced differences in lipid classes and fatty acids profiles between sexes^15,29^, while other studies did not spot statistically significant gender-related discrepancies^5^. Fatty acids profiles of gonads are likely to be related by sea urchin gender, but it is reasonable to believe that such differences would not disprove the aforementioned considerations on lipid storage and metabolism at gut and at gonad level. In particular, the differences in C 18:3 n-3, C 18:2 n-6, C 20:4 n-6 and C 20:5 n-3 found in previous studies between male and female gonads were quite low (maximum 2–4% of total FAME). Gender differences are ascribable to the increasing presence of lipid-rich gametes (oocytes or sperm) during the gonad maturation period. Also differences in lipid classes are expected in this period, being triglycerides mainly present in female gametes^29,56^. According to previous reports, during the reproductive period females of both *P. lividus* and *Arbacia lixula* showed lower proportions of 20:4n-6, while 20:5n-3 was higher in males of *P. lividus* and in females of *A. lixula*^56^. In *P. lividus*, such differences were found to be very limited for 20:4n-6 and 20:5n-3 (0.1% and 1.3%, respectively, between mean values of total FAME percentage)^56^. Also in *Arbacia dufresnii* the differences between male and female intact gonads for 20:4n-6, while 20:5n-3 were found to be not very important, but both fatty acids seemed to be slightly more concentrated in male tissues^29^.

In any case, the present study confirms that during maturation stages of gonads, when their nutritive content decreases^20,22^, C 20:5 (n-3) and C 20:4 (n-6) levels increase, and so does their nutritional quality. C 20:5 (n-3) consumption is in fact associated to reduced risk of several chronic diseases^57^. At the same time, previous reports showed that the best commercial value of sea urchin gonads is before the onset of gametogenesis^20,33^. These results are quite relevant not only because they allow to deepen the knowledge of the metabolic response of sea urchin *P. lividus* to season and diet, but also for both improving echinoculture practices and guiding relevant policies directed to regulate the harvest of wild populations. Changes in the concentration of biochemical components in the gonads of sea urchins impact their sensory quality^20,33,34^. In particular, gonads in their mature stages were described as more bitter^34^ and of lower quality overall^33^ than when they are in the growing stage. On the other hand, gonads in the growing stage reach the highest contents of nutrients (protein, fat, carbohydrates)^20^. Harvest of wild sea urchin during the reproductive time should be avoided, and this is particularly important for an endangered species such as *P. lividus*. Echinoculture could provide sea urchin roe for which the harvest time should be carefully scheduled as a function of analytical quality parameters and based on expected use.

In conclusion, *P. oceanica* and *H. scoparia,* primarily constituted *P. lividus* diet in two contrasting sites within the same geographical area. Green algae, especially *C. cylindracea*, supplemented sea urchin diet in the *P. oceanica* meadow prior to gametogenesis, demonstrating the ability of *P. lividus* to select their diet according to requirements. Total lipid content in gonads changed periodically as a function of gametogenic cycle, being relatively independent from dietary lipid intake and showing a maximum during the growing stage and a minimum in mature gonads. Fatty acid profiles of *P. oceanica* and *H. scoparia* were significantly different from each other throughout the year. C 18:3 (n-3) was the main differential dietary marker in *P. lividus* gonads and gut contents. The main PUFA *of P. lividus* gonads, C 20:5 (n-3) and C 20:4 (n-6) were associated to increased consumption of green algae in *P. oceanica* meadow. LC-PUFA were selectively allocated in gonads as a function of reproductive cycle. Conversion of C 18:3 (n-3) to C 20:5 (n-3) and of C 18:2 (n-6) to C 20:4 (n-6) at gut level cannot be excluded, although further research in this sense is desirable. It is worth to note that harvest is generally allowed in Sardinia during gonad maturation, when main nutrients (lipids, carbohydrates, proteins) are at lowest level and also the sensory quality of roe is low, but gonads are rich in healthy LC-PUFA. Our results suggest that rearing of *P. lividus* would be possible with diets very poor in LC-PUFA given a supplement of this nutrients is provided prior to gametogenesis, when gonads are in the growing/premature stages.

## Materials and methods

### Samples collection

Sea urchin samples were harvested monthly from April 2015 to March 2016 from two marine habitats (which will be also referred to as *sites* in the following), namely a rocky bottom and a *P. oceanica* meadow. Both sites are located in the Su Pallosu bay (E008 25N 40 03) (Fig. S1), in the Sinis Peninsula, on the western coast of Sardinia, Italy. The two sites were characterized by different habitats, made up of different marine species. *P. oceanica* meadow was mainly occupied by a bed of *P. oceanica*, with a dense leaf canopy, grown on a high and fragmented matte (i.e. a thick root-rhizome-sediment layer) on a sandy seabed. The rocky bottom habitat was characterized by several photophilic algal communities, such as *Padina pavonica*, *Dictyota dichotoma*, *H. scoparia*, *Codium* spp., *Laurencia* spp. and *Halimeda tuna*.

Fatty acid profile and lipid content have been characterized to relate *P. lividus* gonads composition to the composition of the collected sea grass and macroalgae, also considering *P. lividus* gut content at different seasons.

### Evaluation of sea urchins diet in the two sites

*H. scoparia* was considered the main species populating the rocky bottom site, since it visibly covered the largest part of the site. In order to understand the potential diet of the sea urchins collected from the rocky habitat, beyond *H. scoparia*, we conducted seasonally (two replicates per season) the scraping of the benthic assemblage covering the marine bottom according to Bianchi et al.^58^ In every sampling date, a 10 × 10 cm frame was placed on three different spots of the bottom and all the species included in it were scraped and collected, and further identified under the microscope.

In the *Posidonia* meadow, a destructive sampling method such as scraping was not feasible. Therefore, every sampling month, the gut content of ten sea urchins living in the meadow was checked. The gut content was spread in a Petri dish for observation under a stereoscopic microscope, and analyzed by the "contact method" described by Jones^59^. The analysis of 100 contacts, each one corresponding to a specific food item, provided a good estimation of the digestive content of a sea urchin. Each food item recognized was assigned to a specific group, as done by Chiantore et al.^23^ and Privitera et al.^60^. The mean percentage of contribution of each group was calculated monthly.

### Hystological study

At each sampling date, one of the five gonads was fixed in 10% formalin, dehydrated, embedded in paraffin and then sectioned at 7 mm by a rotary microtome and stained with Haematoxylin (Sigma Aldrich, St. Louis, MO, USA)/Eosin (Carlo Erba Reactifs, Val De Reuil Cedex, France). Throughout the text, we referred to the nomenclature proposed by Byrne: stage I (recovery); stage II (growing); stage III (premature); stage IV (mature); stage V (partly spawned); stage VI (spent).

### Sample preparation

For every harvest, 20 specimens of *P. lividus* of commercial size (test diameter ≥ 50 mm without spines) were collected from each habitat, together with the putative diets mainly populating the same areas. All the samples were immediately transferred to the laboratory in cool boxes. Sea urchins reached the laboratory still alive. They were dissected and gonads and gut content separately stored. Gonads were removed and pooled together into a Petri dish with all those from the same site at the same sampling date. Similarly, gut contents were sampled while carefully removing any gut tissue, pooled together and stored into a Petri dish. Therefore, one pool of gonads and one pool of gut contents were obtained for each site at each sampling date. Briefly, in total 22 pools of sea urchin gonads and 18 pools of gut contents were obtained, since gonads were not sampled on January 2016 and gut contents were not sampled on April and May 2015 and January 2016. Pools were frozen in liquid nitrogen and stored at − 80 °C until extraction.

*Posidonia oceanica*, from the meadow, and *H. scoparia*, from rocky bottom, were collected from June 2015 to March 2016. The sea grass and macroalgae samples were rinsed with marine water at the sampling site and about 100 g were collected into plastic bags filled with seawater. Rinsing did not remove epiphytes. Once samples arrived at the laboratory, the seawater was poured off and they were frozen in liquid nitrogen and stored at − 80 °C until analysis. A total of 8 pools of *P. oceanica* and 8 pools of *H. scoparia* were obtained, since these samples could not be collected in August 2015 and January 2016.

### Lipid extraction

Frozen gonad pools, gut content pools, *P. oceanica* and *H. scoparia* samples were individually crushed by using a pre-cooled stainless-steel mortar filled with liquid nitrogen. A fine frozen powder was then obtained from each sample. Subsequently, about 20 g for the gonad samples, about 50 g for the gut samples and about 100 g for each sea grass or macroalga sample were freeze-dried (Cinquepascal s.r.l., Milano, Italy, mod. Lio2000P). Lipid extraction was carried out in duplicate on lyophilized powders following the Bligh and Dyer method^61^ modified by Anedda et al.^62^. Due to the different lipid content in the samples, the extraction was performed on about 1.25 g of lyophilized gonads, 3.0 g of lyophilized gut contents and 4.5 g of lyophilized sea grass (macroalgae) using 40 ml, 80 ml and 120 ml of the solvent mixture, respectively. Lipid extracts were finally evaporated to constant weight under a gentle stream of nitrogen gas. The extracted lipids were stored at − 30 °C until methylation and GC analysis. Total lipid contents will be reported in the following as a percentage of dry tissue (dry weight percentage, % D.W.). All chemicals and solvents were of analytical grade and were purchased from Sigma Aldrich (St. Louis, MO, USA).

### GC analysis of fatty acids methyl esters

Fatty acids were methylated with the method proposed by Antongiovanni et al.^63^, modified by Siliani et al*.*^22^. Methylations were performed in duplicate for each lipid extract. Methylated samples were analyzed using the method proposed by Santercole et al*.*^64^ and modified by Siliani et al*.*^22^ by using KOH (2 N) in methanol (Sigma-Aldrich, Saint Louis, Missouri, US) and stirring samples for one minute at room temperature. GC analysis of fatty acid methyl esters (FAME) was carried out in duplicate for each methylated samples. An Agilent 7890A gas chromatograph was used (Agilent Technologies, Wilmington, DE) equipped with a FID detector, split/splitless injection port, an autosampler and a Supelco SP-2560 GC column (100 m × 0.25 mm internal diameter × 0.20 μm film thickness). The system was controlled by the Agilent ChemStation (Version B.04.02) chromatography manager. Each FAME was named using the shorthand nomenclature reported by Köfeler^65^ and it was expressed in the following as a percentage of the total FAME. All chemicals and solvents were of analytical grade and were purchased from Sigma Aldrich (St. Louis, MO, USA).

### Photoperiod and sea water temperature

Photoperiod from April 2015 to March 2016 was determined from the US Navy Observatory data^66^ using the following GPS coordinates for Sinis Peninsula: E008 25 N40 03.

Seawater temperature (SWT) was monitored by a HOBO datalogger (Onset Computer Corporation, Pocasset, Massachusetts, USA) positioned at a depth of 5 m at the rocky bottom site. The datalogger measured temperature regularly once an hour; mean monthly values were then calculated.

### Univariate and multivariate statistical analysis (MVA)

All experimental values are reported as their mean and standard deviation (SD). Statistical differences between single and FA profiles as well as fatty acid categories (i.e. polyunsaturated fatty acids, PUFA, monounsaturated fatty acids, MUFA and saturated fatty acids, SFA) were estimated on all sea grass samples collected from *P. oceanica* meadow and on macroalgae from rocky bottom by using two-way analysis of variance (ANOVA), followed by Bonferroni post-test with a P value of less than 0.05 for the rejection of the null hypothesis. Univariate statistical analyses were performed by using GraphPad Prism 5.03 (GraphPad Software Inc., La Jolla, CA, USA).

Additionally, the full FAME profiles of selected sea grass and macroalgae, of sea urchin gonads and gut contents, obtained throughout the year, were imported to SIMCA-P v.13 software (Umetrics Inc., Kinnelon, NJ, USA) for MVA purposes (“Supplementary Material”).

### Ethics declaration

All applicable international, national and/or institutional guidelines for the care and use of animals were followed.

## Supplementary Information

Supplementary Information.

## Data Availability

The datasets generated during the current study are available from the corresponding author on reasonable request.
