# Supplementary material for: Lipid metabolism of sea urchin Paracentrotus lividus in two contrasting natural habitats
Source: Sci Rep. 2021 Jul 8;11:14174. doi: 10.1038/s41598-021-93669-9 (PMC8266866; doi:10.1038/s41598-021-93669-9)
Supplement: Supplementary file 1 — Supplementary Information. [file 41598_2021_93669_MOESM1_ESM.docx]

**Supplementary Material**

## Lipid metabolism of sea urchin *Paracentrotus lividus* [in two contrasting natural habitats](http://www.plantphysiol.org/content/69/4/757.short)

Roberto Anedda ^a,*^, Silvia Siliani ^a^, Riccardo Melis ^a^, Barbara Loi ^b^ , Maura Baroli ^b^

^a^ Porto Conte Ricerche S.r.l. – S.P. 55 Porto Conte – Capo Caccia, Km 8.400, Loc. Tramariglio, Alghero (SS) – Italy

^b^ IMC - International Marine Centre, Loc. Sa Mardini, 09170 Torregrande (OR) – Italy

**Roberto Anedda. Corresponding author.** anedda@portocontericerche.it,

phone +39 079 998578, fax +39 079 998567.

**Silvia Siliani** silvia.siliani@gmail.com

**Riccardo Melis** melis@portocontericerche.it

**Barbara Loi** b.loi@fondazioneimc.it

**Maura Baroli** m.baroli@fondazioneimc.it

**Table S1.** List of the species of the benthic assemblage in the rocky habitat. Each taxa was identified through the scraping method, during seasonal samplings (2 dates per season), using a 10x10 cm frame (triplicate).

| **Rhodophyta** | **Ochrophyta** | **Chlorophyta** | **Magnoliophyta** |
| --- | --- | --- | --- |
| *Acrothamnion preissii* | *Acinetospora crinita* | *Anadyomene stellata* | *Posidonia oceanica* |
| *Amphiroa cryptarthrodia* | *Cladosiphon cylindricus* | *Bryopsis duplex* |  |
| *Amphiroa rigida* | *Cutleria multifida* | *Chaetomorpha sp.* |  |
| *Antithamnion amphigeneum* | *Cystoseira compressa* | *Cladophora sp.* |  |
| *Apoglossum ruscifolium* | *Dictyota dichotoma* | *Codium effusum* |  |
| *Asparagopsis taxiformis* | *Ectocarpus siliculosus* | *Codium vermilara* |  |
| *Bonnemaisonia asparagoides* | *Feldmannia lebelii* | *Dasycladus vermicularis* |  |
| *Callithamnion caudatum* | *Halopteris filicina* | *Flabellia petiolata* |  |
| *Callithamnion tetragonum* | *Halopteris scoparia* | *Halimeda tuna* |  |
| *Ceramium tenerrimum* | *Padina pavonica* | *Pseudochlorodesmis furcellata* |  |
| *Ceramium virgatum* | *Sargassum vulgare* | *Valonia sp.* |  |
| *Champia parvula* | *Sphacelaria cirrosa* |  |  |
| *Chondria sp.* | *Sphacelaria plumula* |  |  |
| *Corallina elongata* | *Taonia atomaria* |  |  |
| *Corallinaceae (encrusting)* | *Zanardinia typus* |  |  |
| *Cottoniella filamentosa* |  |  |  |
| *Crouania attenuata* |  |  |  |
| *Dasya hutchinsae* |  |  |  |
| *Dasya rigidula* |  |  |  |
| *Delesseriaceae (not identified)* |  |  |  |
| *Eupogodon planus* |  |  |  |
| *Eupogodon spinellus* |  |  |  |
| *Gayliella flaccida* |  |  |  |
| *Gelidium pulchellum* |  |  |  |
| *Gelidium pulsillum* |  |  |  |
| *Griffithsia phyllamphora* |  |  |  |
| *Gulsonia nodulosa* |  |  |  |
| *Halimenia elongata* |  |  |  |
| *Haliptilon virgatum* |  |  |  |
| *Halopithys incurva* |  |  |  |
| *Halydictyon mirabile* |  |  |  |
| *Herposiphonia secunda* |  |  |  |
| *Heterosiphonia crispella* |  |  |  |
| *Hypnea spinella* |  |  |  |
| *Hypoglossum hypoglossoides* |  |  |  |
| *Irvinea boergesenii* |  |  |  |
| *Jania rubens* |  |  |  |
| *Laurencia complex* |  |  |  |
| *Lomentaria articulata* |  |  |  |
| *Monosporus pedicellatus* |  |  |  |
| *Nemalion helminthoides* |  |  |  |
| *Peyssonnelia rubra* |  |  |  |
| *Pleonosporium borreri* |  |  |  |
| *Polysiphonia flocculosa* |  |  |  |
| *Polysiphonia opaca* |  |  |  |
| *Polysiphonia sertularioides* |  |  |  |
| *Polysiphonia subulifera* |  |  |  |
| *Pterothamnion plumula* |  |  |  |
| *Seirospora giraudyi* |  |  |  |
| *Sphondylothamnion multifidum* |  |  |  |
| *Spyridia filamentosa* |  |  |  |
| *Womersleyella setacea* |  |  |  |
| *Wrangelia penicillata* |  |  |  |

**Table S2.** Mean percent contribution (± standard deviation) of the main taxa identified in the gut contents of sea urchins living in the *P. oceanica* meadow. For each sampling month, N=10 sea urchins were dissected. Fu = Fucales; Di = Dictyotales; ErBr = Erected brown algae; ErGr = Erected green algae; ErRe = Erected red algae; Tu = Turf species; ArCo = Articolated corallines; EnCo = Encrusting corallines; EnAl = Encrusting algae (not corallines); Po = Posidonia oceanica; An = Animal taxa; nf = no items of that category were found.

| **Taxa** | *Apr15* | *May15* | *Jun15* | *Jul15* | *Aug15* | *Sep15* | *Oct15* | *Nov15* | *Dec15* | *Jan16* | *Feb16* | *Mar16* |
| --- | --- | --- | --- | --- | --- | --- | --- | --- | --- | --- | --- | --- |
| *Fu* | nf | nf | nf | 0.4±1.3 | 0.1±0.3 | nf | nf | nf | nf | nf | nf | 1.3±3.4 |
| *Di* | nf | 8.3±4.7 | 8.9±8.2 | 1±1.3 | 0.8±1.0 | 0.1±0.3 | 2.4±1.9 | 4.2±5.5 | 0.3±0.7 | 7.0±4.1 | 5.2±3.2 | 9.8±5.8 |
| *ErBr* | 2.4±4.6 | 7.6±3.3 | 9.2±7.5 | 4±3.9 | 5.5±5.5 | 3.8±3.1 | 4.1±4.5 | 3.1±2.6 | 3.8±2.3 | 3.3±2.5 | 3.8±2.8 | 7.2±4.8 |
| *ErGr* | 0.2±0.4 | 1.3±1.4 | 2.1±3.4 | 3.8±4.0 | 1.5±2.0 | 0.7±1.3 | 10.8±12.8 | 15.1±6.8 | 24.5±17.7 | 6.4±5.6 | 4.5±4.5 | 4.8±3.5 |
| *ErRe* | 3.4±3.2 | 8.9±4.8 | 2.5±1.3 | 1.3±1.6 | 2.3±1.8 | 2.6±2.9 | 2.5±2.4 | 7.2±10.2 | 2.8±2.3 | 11.3±4.2 | 4.1±1.9 | 4.2±4.0 |
| *Tu* | 43.3±6.8 | 31.7±4.2 | 17.2±9.2 | 27.3±10.9 | 24.3±8.7 | 18.7±7.3 | 22.3±11.7 | 11.9±3.0 | 12.9±5.3 | 16.6±6.5 | 10.5±3.1 | 16.1±5.5 |
| *ArCo* | 0.3±0.5 | 3.6±2.2 | 1.4±1.8 | 0.3±0.7 | nf | nf | 1.1±1.3 | 3.6±3.0 | 1.7±2.5 | 3.8±2.0 | 2.1±1.6 | 1.5±1.7 |
| *EnCo* | 1.8±2.3 | 3.9±2.5 | 3.1±2.2 | 7±3.6 | 10.3±4.3 | 10.6±3.7 | 13.1±5.0 | 7.7±3.2 | 8.7±4.5 | 7.5±1.4 | 11.6±3.6 | 7.8±3.1 |
| *EnAl* | 3.2±4.3 | 0.9±1.2 | 0.8±1.9 | 0.9±2.8 | 0.3±0.9 | nf | 1.2±1.8 | 0.8±1.2 | 2.9±3.4 | 1.5±2.0 | 2.4±3.2 | 2.4±3.0 |
| *Po* | 43.2±3.5 | 26.4±4.9 | 48.5±6.3 | 48.7±6.4 | 53.1±7.2 | 63.3±6.8 | 42.2±8.8 | 46.3±9.9 | 42.2±15.5 | 42.3±7.9 | 55.2±7.0 | 44.7±7.7 |
| *An* | 2.2±1.5 | 7.4±3.9 | 6.3±8.4 | 5.3±3.9 | 1.8±3.0 | 0.2±0.4 | 0.3±0.9 | 0.1±0.3 | 0.2±0.4 | 0.3±0.5 | 0.6±0.8 | 0.2±0.4 |

**Figure S1.** Mean percent contribution of the main taxa identified in the gut contents of sea urchins living in the *P. oceanica* meadow. For each sampling month, N=10 sea urchins were dissected. Fu = Fucales; Di = Dictyotales; ErBr = Erected brown algae; ErGr = Erected green algae; ErRe = Erected red algae; Tu = Turf species; ArCo = Articolated corallines; EnCo = Encrusting corallines; EnAl = Encrusting algae (not corallines); Po = Posidonia oceanica; An = Animal taxa.

**Table S3.** Results from fitting total lipids of gonads, photoperiod and sea water temperature. Summary of the best estimated parameters, calculated according to the equation 1 with a fixed wavelength *w*: *y0* vertical shift, *A* amplitude shift and *φ* phase shift, with standard errors (SE) and fit goodness index, R^2^, with related root mean square errors (RMSE).

| **Models** | ***y0*** | ***A*** | *φ* | ***w*** | **R^2^** |
| --- | --- | --- | --- | --- | --- |
| **Photoperiod (light hours)** | 12.210 (0.021) | 2.754 (0.030) | 31.054 (0.619) | 365.242 | 0.999 (0.070) |
| **Sea water temperature (°C)** | 18.543 (0.415) | 5.828 (0.609) | -30.851 (5.648) | 365.242 | 0.921 (1.360) |
| **Total lipids (%D.W.) of gonads from *P. oceanica* meadow** | 15.918 (0.401) | 2.159 (0.597) | -21.969 (14.501) | 365.242 | 0.622 (1.310) |
| **Total lipids (%D.W.) of gonads from rocky bottom** | 15.712 (0.168) | 0.900 (0.235) | -53.744 (15.595) | 365.242 | 0.650 (0.551) |

**Comment to Table S3:** Experimental data were fitted according to the model proposed by Siliani et al. (Siliani, S. *et al.* (2016), *Mar. Environ. Res.* 113, 124–133).

Briefly, the following model (**Equation** (**1**)) was used to fit the experimental data including total lipid content of gonads, photoperiod and seawater temperature

**Equation (1)** $y=y0+A\sin\left(\pi\frac{x+\varphi}{\frac{w}{2}}\right)$

where *y0* is the vertical shift, *A* is the phase amplitude, *φ* is the phase shift and *w* is the period, which refers to an average solar year duration, fixed at 365,242 days. Fitting of experimental data was carried out by means of the Statistic Toolbox™ of Matlab R2020b version (The Mathworks Inc., Natick, MA, USA).

Fittings associated to *P. lividus* gonads sampled either from *P. oceanica* meadow or rocky bottom show some differences. Values of the amplitude of the sine wave curve *A*, showed that the lipid content changed to a larger extent in gonads from *P. oceanica* meadow than from rocky bottom (i.e. reached a higher maximum and a lower minimum). The time-displacement of the sine curve *φ*, showed that the maximum lipid content was reached approximately 30 days later in gonads from rocky bottom than from *P. oceanica* meadow, minimum values being correspondingly shifted. With respect to photoperiod and seawater temperature, it can be noted that the *φ* of photoperiod exceeded of 53 and 85 days the *φ* of total lipids in *P. oceanica* meadow and rocky bottom, respectively. On the other hand, *φ* of seawater temperature was 23 days shorter than the *φ* of total lipids in *P. oceanica* meadow and it was 9 days longer than the *φ* of total lipids in rocky bottom.


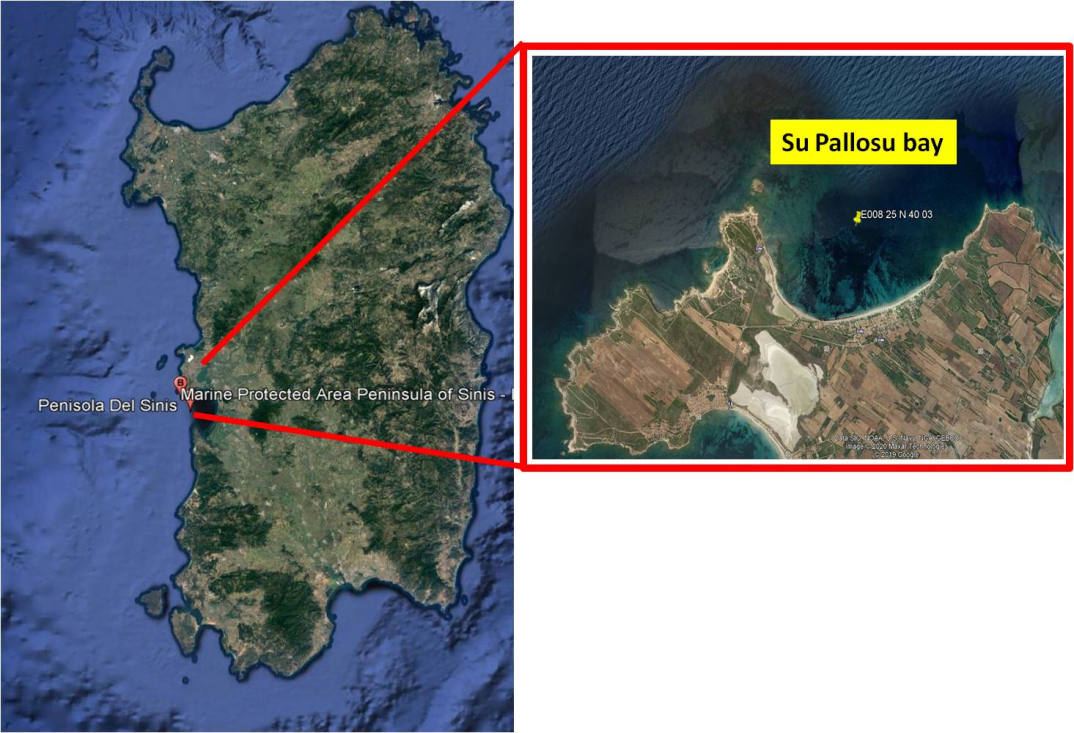


**Figure S2**. Location of the sites where the effect of the two habitats on the sea urchin fatty acid profiles has been studied. Maps were generated by using Google Maps (Google. (n.d.). Sardinia, Italy; Su Pallosu (OR, Sardinia, Italy). Retrieved from https://www.google.com/maps/place/Sardegna/) and successfully modified by using Microsoft Office Power Point 2007 version (Microsoft Corporation (2007)).

**Figure S3**. Annual variation of fatty acid classes PUFAs, MUFAs and SFAs of sea grass and macroalgae (a), gut contents (b) and gonads (c) in *P. lividus* from *P. oceanica* meadow and rocky bottom. Means of each lipid classes, within each site, sharing same letters stand for not significant differences (P > 0.05).

Multivariate Statistical Analysis (MVA) of fatty acid profiles

FAME profiles of selected sea grass and macroalgae, of sea urchin gonads and gut contents, obtained throughout the year, were imported to SIMCA-P v.13 software (Umetrics Inc., Kinnelon, NJ, USA). Preliminary data centering and pareto scaling were initially applied before the MVA models construction and validation steps. In order to examine the datasets for outliers and to give a first overview of samples patterns, unsupervised principal component analysis (PCA) was applied[1]. Orthogonal partial least square discriminant analysis (OPLS-DA) models were then built to emphasize the PCA observed clustering, by using more robust supervised approach[2]. Additionally, from the OPLS-DA modelling, the FAMEs that significantly contributed to groups clustering were identified by means of variable predicted importance (VIP) scores, with the cut-off value set at 1. Goodness of all MVA models was evaluated for quality and reliability by the cumulative R2X (cum) and the predictive R2Y (cum) and Q2 (cum) cross-validation parameters. A limit threshold of Q2 (cum) >0.5 was adopted to validate the model.[3,4].

MVA inspection of fatty acids profiles, based on PCA and OPLS-DA models, allows to better underline the contribution of each fatty acid in the discrimination of samples from different habitats.

MVA evidenced a clear discrimination of the fatty acid profiles in *P. oceanica* and *H. scoparia*, gut contents and gonads (**Fig. S4, S5**). All MVA models have been characterized by an acceptable goodness parameter (Q^2^ (cum) > 0.5), the highest values being registered for the discrimination between *P. oceanica* and *H. scoparia* (Q^2^ (cum) = 0.87 in PCA and 0.98 in OPLS-DA). All supervised OPLS-DA models were built based on the good discriminative power of preliminary PCA and were characterized by very high predictive ability (Q^2^ (cum) > 0.85).

It was found that the most discriminant fatty acid according to OPLS-DA VIP ordered scores was C 18:3 (n-3) (**Tab. S4**), thus confirming previous univariate appraisal (**Fig. 3** in Main text).

In descending order of importance according to VIP values, C 18:3 (n-3) and C 14:0 were followed by other SFA: C 21:0 in gonads and gut contents, and C 16:0 in the sea grass and macroalgae analyzed (**Tab. S4**).

C 14:0 is recognized by MVA as a key fatty acid in discriminating the lipid profile of marine macrophytes, gut contents and gonads from the two habitats (**Fig. 3** in Main text**, Tab. S4**). Likely, the discriminant ability of C 14:0 is due to the low percentage of this FA in *P. oceanica*[5] with respect to *H. scoparia*[6].

While it was found that MUFA mainly affect the differences between sea urchin gonads collected in different growing sites[7], in the present study the sampling sites rested on the same geographical area (Sinis Peninsula), and shared very similar environmental conditions. Therefore the significant differences in C 18:3 (n-3) should be mainly ascribed to sea urchin diets in the two habitats. It is also noteworthy that C 18:2 (n-6) was present in all the OPLS-DA VIP lists with values higher than 1.4 (**Tab. S4**), thus highlighting its decisive role in discriminating samples from different habitats.

**Table S4**- FAME-related VIP scores derived from the OPLS-DA classification according to habitat (*P. oceanica* meadow vs. rocky bottom) discrimination. Only variables with VIP score >1 are reported.

| **HABITAT DISCRIMINATION (*P. oceanica* meadow vs. rocky bottom)** | | | | | |
| --- | --- | --- | --- | --- | --- |
| ***P. oceanica* and *H. scoparia*** | | **Gut contents** | | **Gonads** | |
| **FAME** | **OPLS-DA VIP** | **FAME** | **OPLS-DA VIP** | **FAME** | **OPLS-DA VIP** |
| C 18:3 (n-3) | 4.21 | C 18:3 (n-3) | 3.65 | C 18:3 (n-3) | 3.41 |
| C 14:0 | 2.24 | C 14:0 | 2.27 | C 14:0 | 2.01 |
| C 16:0 | 1.91 | C 21:0 | 2.08 | C 21:0 | 2.01 |
| C 18:2 (n-6) | 1.85 | C 18:2 (n-6) | 2.07 | C 20:1 (n-15) | 1.70 |
| C 16:1 (n-7) | 1.71 | C 20:2 (n-6) | 1.50 | C 20:2 (n-6) | 1.57 |
| C 20:4 (n-6) | 1.50 | C 16:1 (n-5) | 1.34 | C 18:2 (n-6) | 1.49 |
| C 20:5 (n-3) | 1.28 | C 20:1 (n-9) | 1.18 | C 16:1 (n-5) | 1.46 |
| C 18:1 (n-9) | 1.22 | C 20:5 (n-3) | 1.18 | C 20:5 (n-3) | 1.39 |
|  |  | C 20:1 (n-15) | 1.10 | C 16:1 (n-7) | 1.33 |
|  |  |  |  | C 22:1 (n-11) + (n-9) | 1.29 |
|  |  |  |  | C 20:1 (n-9) | 1.06 |
|  |  |  |  | C 18:1 (n-7) | 1.03 |


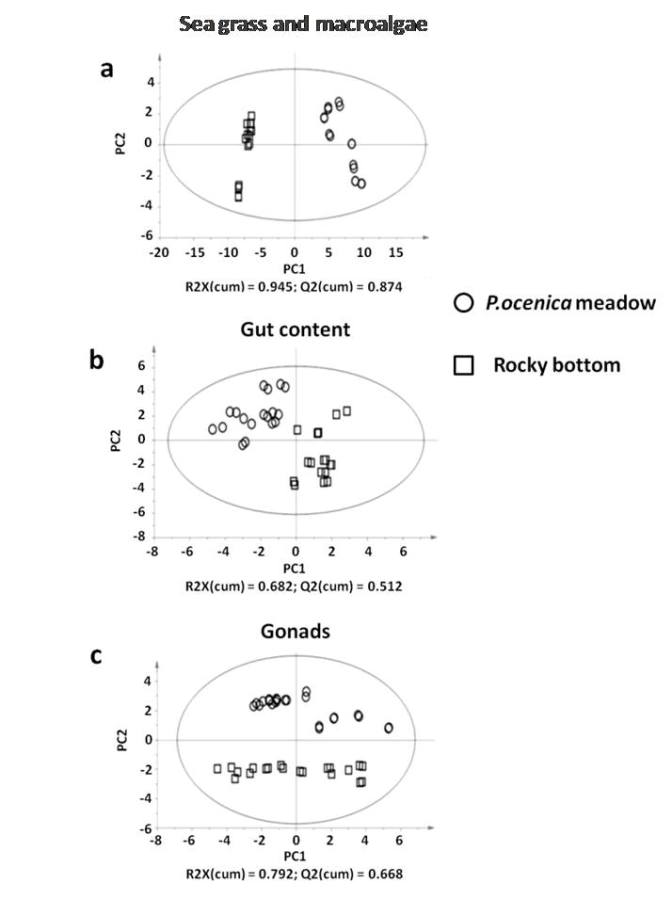


**Figure S4**. PCA scores plot based on the FAMEs profiles of sea grass and macroalgae (a) and *P. lividus* gut contents (b) and gonads (c) collected over one year from *P. oceanica* meadow (circles) and rocky bottom (box). Related cross-validation parameters R2X (cum) and Q2 (cum) are reported at the bottom of each graph. The black ellipses drawn represent T2 Hotelling's plots with 95% confidence.


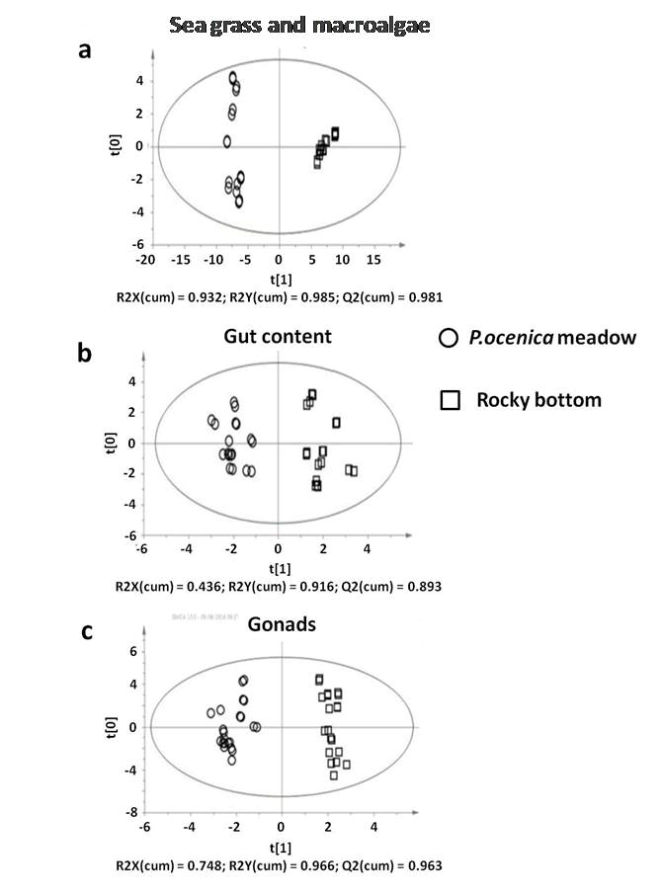


**Figure S5.** OPLS-DA scores plot based on the FAME profiles of *P. lividus* from sea grass and macroalgae (a) and *P. lividus* gut contents (b) and gonads (c) collected over one year from *P oceanica* meadow and rocky bottom. Related cross-validation parameters R2X (cum), R2Y (cum) and Q2 (cum) are reported at the bottom of each graph. The black ellipses drawn represent T2 Hotelling's plots with 95% confidence.

**References**

1. Jolliffe, I. Principal Component Analysis. In *International Encyclopedia of Statistical Science*; Lovric, M., Ed.; Springer Berlin Heidelberg: Berlin, Heidelberg, 2011; pp. 1094–1096 ISBN 978-3-642-04898-2.

2. Trygg, J.; Holmes, E.; Lundstedt, T. Chemometrics in metabonomics. *J. Proteome Res.* **2007**, *6*, 469–79, doi:10.1021/pr060594q.

3. Wiley: Multivariate Calibration - Harald Martens, Tormod Næs Available online: http://eu.wiley.com/WileyCDA/WileyTitle/productCd-0471930474.html (accessed on Feb 7, 2014).

4. Eastment, H.T.; Krzanowski, W.J. Cross-Validatory Choice of the Number of Components From a Principal Component Analysis. *Technometrics* **1982**, *24*, 73–77, doi:10.1080/00401706.1982.10487712.

5. Viso, A.C.; Pesando, D.; Bernard, P.; Marty, J.C. Lipid components of the mediterranean seagrass Posidonia oceanica. *Phytochemistry* **1993**, *34*, 381–387, doi:10.1016/0031-9422(93)80012-H.

6. Campos, A.M.; Matos, J.; Afonso, C.; Gomes, R.; Bandarra, N.M.; Cardoso, C. Azorean macroalgae (Petalonia binghamiae, Halopteris scoparia and Osmundea pinnatifida) bioprospection: a study of fatty acid profiles and bioactivity. *Int. J. Food Sci. Technol.* **2019**, doi:10.1111/ijfs.14010.

7. Siliani, S.; Melis, R.; Loi, B.; Guala, I.; Baroli, M.; Sanna, R.; Uzzau, S.; Roggio, T.; Addis, M.F.; Anedda, R. Influence of seasonal and environmental patterns on the lipid content and fatty acid profiles in gonads of the edible sea urchin Paracentrotus lividus from Sardinia. *Mar. Environ. Res.* **2016**, *113*, 124–133, doi:10.1016/j.marenvres.2015.12.001.
